# Severity analysis of crashes involving in-state and out-of-state large truck drivers in Alabama: A random parameter multinomial logit model with heterogeneity in means and variances

**DOI:** 10.1016/j.heliyon.2022.e11989

**Published:** 2022-11-30

**Authors:** Sunday Okafor, Emmanuel Kofi Adanu, Steven Jones

**Affiliations:** a Department of Civil, Construction, and Environmental Engineering, The University of Alabama, USA; b Alabama Transportation Institute, USA

**Keywords:** Large truck safety, In-state drivers, Out-of-state drivers, Fatigue, Random parameter modelling

## Abstract

The trucking sector contributes significantly to the economic vitality of the United States. Large trucks are primarily used for transporting goods within and across states. Despite its economic importance, large truck crashes constitute public safety concerns. To minimize the consequences, there is a need to understand the factors that contribute to the severity outcomes of truck-involved crashes. Since many large truck drivers transport goods across several states, the driver-centered crash factors are expected to differ between in-state and out-of-state drivers. For this reason, this study developed two random parameters multinomial logit models with heterogeneity in means and variances to examine the factors contributing to the severity of crashes involving in-state and out-of-state large truck drivers in Alabama. The study was based on the 2016–2020 large truck crashes in Alabama. After data cleaning and preparation, it was observed that approximately 20% of in-state and 23% of out-of-state large truck crashes were fatigue-related. There were more speeding related crashes (12.4%) among in-state large truck drivers, but the contribution of speeding to crash severity outcomes was only significant in the out-of-state model. More crashes related to red light running violation (14.2%) were observed among out-of-state drivers, pointing to the fundamental issues of fatigue and unfamiliarity with the operations of signalized intersections in Alabama. The study contributes to the literature on large truck crashes by uncovering the nuances in crashes involving in-state and out-of-state large truck drivers. Despite the seeming similarity in factors that influence crash outcomes, this study provides the basis for truck drivers’ training and communication campaigns on the differences that may exist in roadway characteristics from state to state. Also, policy formulations and strategies that prioritizes the well-being of the large truck drivers and creates a better working condition for them should be explored.

## 1 Introduction

The trucking sector contributes significantly to the economic vitality of the United States. In 2020, over 900,000 active truck drivers were in the sector, generating approximately $732 billion in revenue and representing 1% of the country's annual revenue (Duffin, 2022; Placek, 2022). The sector relies primarily on large trucks to transport goods within and across different states. The outbreak of COVD-19 in 2020 and the following lockdown did not significantly impact the trucking industry as its regarded as an essential service. Despite the economic importance of the trucking sector, large truck (with a gross vehicle weight rating greater than 10,000 pounds) crashes constitute public safety concerns. These vehicle types represent 4% of all registered vehicles but contribute about 9% to fatal crashes (National Safety Council, 2022; NHTSA, 2022a). Occupants of other vehicles and non-vehicle occupants (like pedestrians and bicyclists) are more vulnerable to severe or fatal injury in crashes involving large trucks (NHTSA, 2022b).

Moreover, large truck crashes often result in severe and fatal injuries compared to other vehicle crashes due to their size and weight (Behnood and Mannering, 2019; Liu and Fan, 2022). Data from the National Safety Council (National Safety Council, 2022) showed that fatalities in large truck crashes increased between 2014 and 2019, with 70% of the fatalities representing occupants of other vehicles. Despite the decrease in large truck-related fatalities observed in 2020 compared to the previous year, the number of non-vehicle occupants (like pedestrians and bicyclists) killed increased by 9.1% (NHTSA, 2022b). These statistics demonstrate the need to mitigate large truck crashes and their impact on public safety by identifying the significant contributing factors to implement effective countermeasures.

Aside from the public health safety concerns associated with large truck crashes, the stressful working conditions among professional truck drivers encourage unhealthy lifestyles resulting in higher risks of chronic diseases (Garbarino et al., 2018). Compared to the population average, the life expectancy of male truck drivers in the U.S is 16.1% and 25.8% lower for unionized and independent drivers, respectively (Apostolopoulos et al., 2010). Long-haul routes expose truck drivers to various mental health-related risks associated with the transportation environment (Apostolopoulos et al., 2010). In most cases, truck drivers spend consecutive days away from their families and engage in stimulants, alcohol, and drug use to meet the high delivery requirements (Heaton, 2005). These enormous industry demands often result in stress and health challenges for truck drivers and can significantly impact road safety. Therefore, understanding the peculiar challenges of truck drivers will inform more effective countermeasures that complement the results of large truck-involved crash models.

Previous studies have examined the association between large truck injury severity outcomes and their contributing factors with a diverse focus (Alrejjal et al., 2021; Azimi et al., 2019, 2020; Behnood and Mannering, 2019; Hosseinzadeh et al., 2021; Liu and Fan, 2022; Tahfim and Yan, 2021). Azimi et al. (2019) conducted a severity analysis of large truck crashes in Florida between 2007 and 2017 using a random parameter ordered logit model. They found that vision obstruction, running red light, and following too close increased crash severity significantly. By developing a mixed logit model as a baseline compared to selected machine learning models, Li et al. (2020) examined the factors contributing to the severity of large truck crashes in Texas using data from the Texas Crash Records Information System between 2011 and 2015. They identified that driving under the influence of drugs or alcohol and fatigue were the most significant factors contributing to the severity outcomes. They also found that the presence of curbs and medians and lanes and shoulders with adequate widths can prevent severe large truck crashes. Pulugurtha et al. (2022) study of truck-involved crashes in North Carolina revealed that fatigue, inattention, and impairment are the driver-related factors contributing significantly to crash occurrence.

Some other studies considered specific crash types like rollover or run-off-road crashes and single-vehicle or multi-vehicle crashes. For instance, Azimi et al. (2020) conducted a severity analysis of large truck rollover crashes in Florida using a random parameter ordered logit model and identified lighting condition, time of the crash, and driver vision obstruction as significant contributing factors. Also, Liu & Fan (2022) study of rear-end large truck crashes in North Carolina found that driving under the influence of drugs or alcohol, rural roadways, dark lightning condition, grade roadway configuration, and speed limits above 50 mph increased injury severity significantly. Adanu et al., 2021a, Adanu et al., 2021b, on the other hand, considered the injury severity of lane change crashes involving commercial motor vehicles on interstate highways. The study showed that lane changing crashes on unlit roadway and involving older drivers, at-fault commercial vehicles, and female drivers are more likely to result in major injury.

Spatio-temporal differences like time of the day or location (urban/rural areas, interstates, intersections) have also been explored among large truck-involved crashes. For instance, Pahukula et al. (2015) conducted a time-of-the-day analysis of crashes involving large trucks in urban areas using reported data by Texas Peace Officer's Crash Reports. For the analysis, they separated the data into five time periods and found seatbelt use, sideswipe collision, driver age less than 25, and male drivers to be significant factors in each period. For large truck crashes on mountainous interstates, Alrejjal et al. (2021), used correlated random parameters binary logit model and found that strong winds, overcorrections, and run-off-road increased the risk of rollover. Thereby, increasing the possibility of a severe crash outcomes. Additionally, Islam et al. (2014) developed four different random parameter logit models to comprehensively analyze single and multi-vehicle large truck at-fault crashes on rural and urban roadways in Alabama. Their results revealed that driver fatigue, wet surface, overtaking, and hitting fixed objects contributed significantly to single and multi-vehicle crashes in rural areas. These studies identified several factors contributing to the severity of large truck-related crashes given different circumstances.

Despite the studies uncovering the association between explanatory variables and severity outcomes of crashes involving large trucks, no known research effort has explored crashes involving in-state and out-of-state large truck drivers. Viewing large truck crashes from the lens of the drivers' primary state of residence is important to understand whether familiarity with driving in a state plays a role in the types of crashes that involve truck drivers. Indeed, drivers licensed and residing within the state are more likely to be familiar with the state's driving environment than drivers from other states. Similar factors might contribute differently to the severity of crashes involving in-state and out-of-state large truck drivers. It is imperative to capture such similarities and differences to be able to target driver-centered countermeasures.

Familiarity with the road network and traffic conditions influence driving styles and driving behaviors. Generally, in-state drivers are familiar with the local context of the transportation system. This knowledge, or lack thereof on the part of out-of-state drivers, influences traffic safety. This study aims to identify differences in the contributing factors to large truck crashes in Alabama involving in-state and out-of-state drivers and proposes some countermeasures. To achieve this, separate random parameters multinomial logit models with heterogeneity in means and variances were developed to identify the explanatory variables associated with crashes involving in-state and out-of-state large truck drivers in Alabama. Five-year (2016–2020) crash data from the Critical Analysis Reporting Environment (CARE) developed by the University of Alabama Center for Advanced Public Safety were used for the analysis.

## 2 Data description

**Figure 1:**
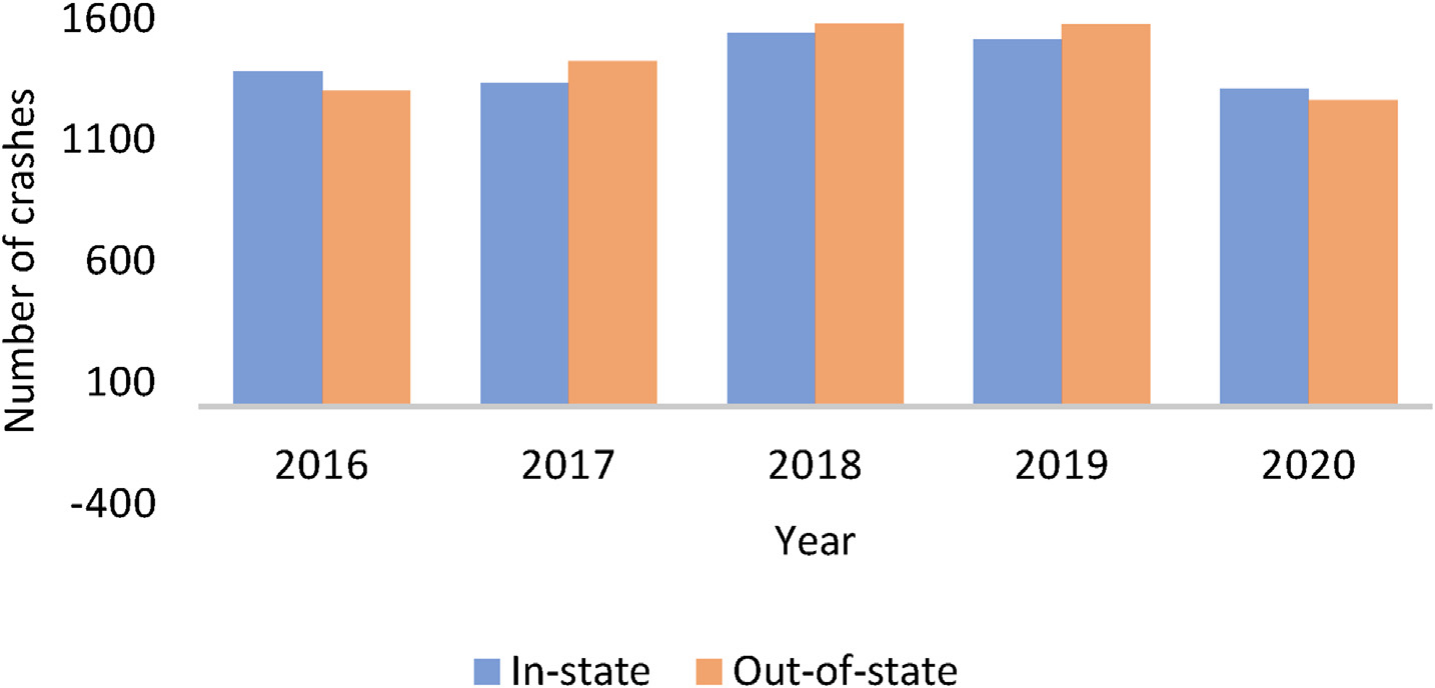
Frequency of large truck crashes over the study period.

The study data was extracted from the Critical Analysis Reporting Environment (CARE) developed by the University of Alabama Center for Advanced Public Safety. Data from 2016 to 2020 was obtained, representing the most recent 5-year records of large truck crashes. The data contained five crash severity levels classified on the KABCO scale, where K = fatal injury, A = incapacitating injury, B = evident injury, C = possible injury, and O = no injury. We reclassified K and A as severe injury (SI), B and C as minor injury (MIN), and O as no injury (NI). Crashes involving large trucks licensed in Alabama were separated as in-state crashes and other trucks as out-of-state crashes. A total of 6943 in-state and 6863 out-of-state large truck-involved crash observations were used for the modeling. Figure 1 shows the number of crashes over the study period. It indicates an increasing trend from 2016 to 2019 and a decrease in 2020. There were more in-state crashes in 2016 and 2020 than out-state crashes. The reduction in crashes observed in 2020 can be related to the Covid-19 pandemic when traffic volume decreased. However, the proportion of severe crashes in 2019 and 2020 are similar for both in-state and out-of-state crashes despite the reduction in total crashes in 2020.

**Figure 2:**
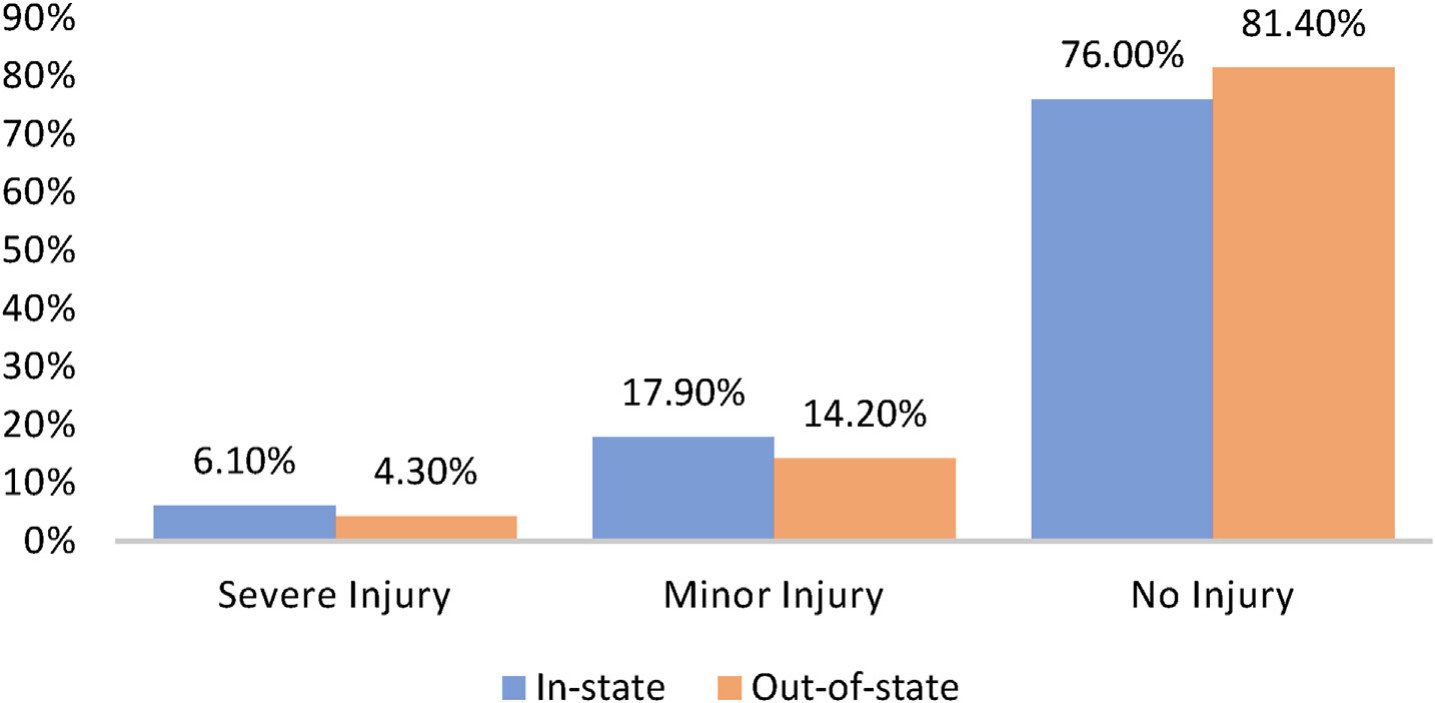
Proportion of in-state and out-of-state large truck crash severity.

Figure 2 shows the proportion of in-state and out-of-state crashes for severe, minor, and no injury. In-state crashes resulted in a higher proportion of severe and minor crash severity, and out-of-state crashes resulted in a higher proportion of no injury crash severity.

**Figure 3:**
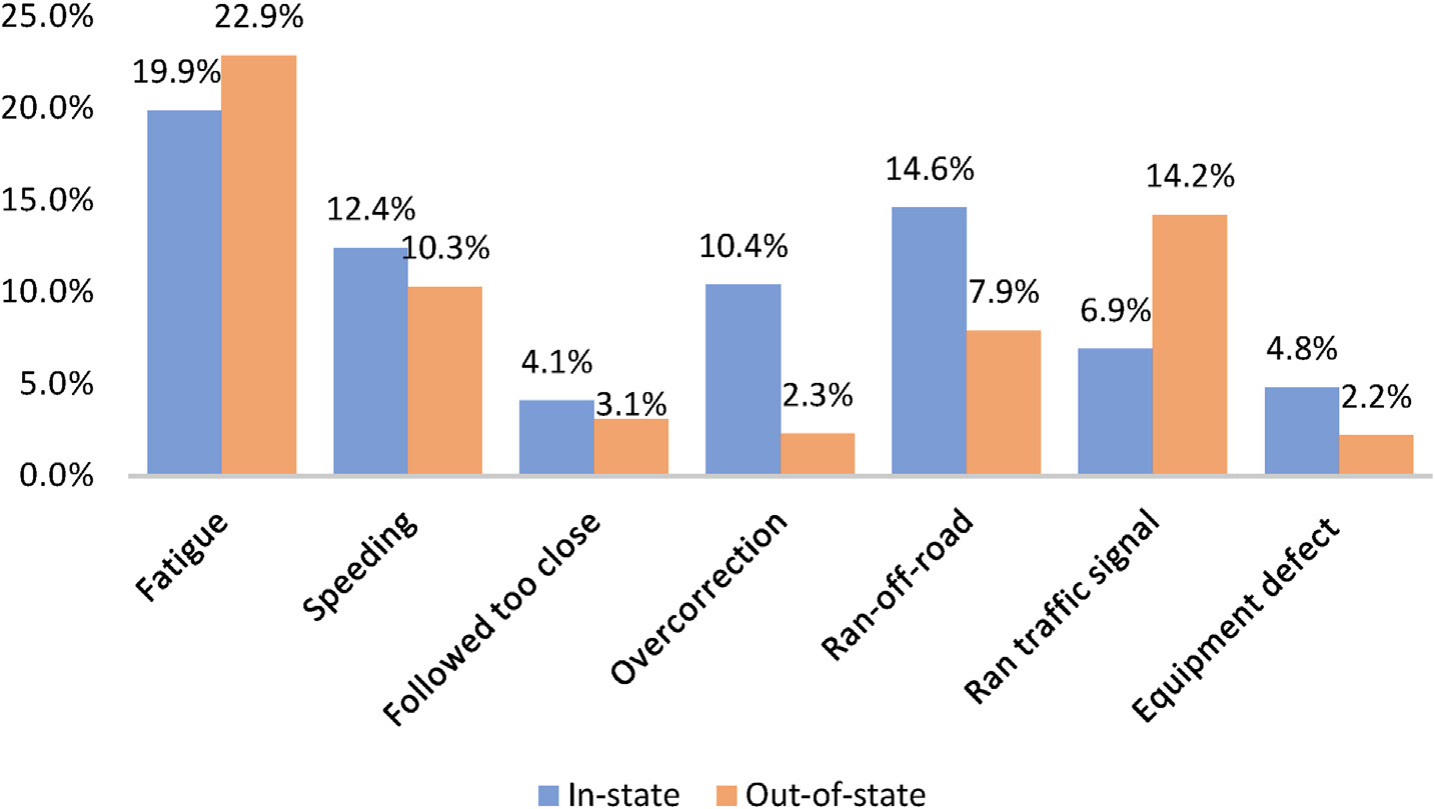
Selected primary contributing factors for in-state and out-of-state severe crashes.

Figure 3 gives the proportions of selected primary contributing factors to severe crashes. “Fatigue” and “ran traffic light” contributed 22.9% and 14.2% to out-of-state major crashes, respectively, higher than the in-state crash observations. Overcorrection and run-off-road contributed more to in-state severe crashes than out-of-state severe crashes.

Tables 1 and 2 present the detailed descriptive statistics of the in-state and out-of-state large truck crash variables used in the model estimation.

**Table 1:** Descriptive statistics of selected in-state large truck crash variables.

Variable	Severe Injury		Minor Injury		No Injury		Total
	Count	Percent	Count	Percent	Count	Percent	Count
*Driver age*							
Less than 25 years old	14	6.1%	47	20.6%	167	73.2%	228
25–40 years old	120	5.8%	367	17.8%	1580	76.4%	2067
41–65 years old	240	5.8%	737	17.9%	3145	76.3%	4122
Above 65 years old	47	9.0%	95	18.2%	381	72.8%	523
*Driver race*							
Caucasian	290	6.7%	781	18.1%	3249	75.2%	4320
Black	125	4.9%	448	17.5%	1983	77.6%	2556
Others	20	7.5%	71	26.7%	175	65.8%	266
*Crash location*							
Intersection	137	4.1%	564	17.0%	2624	78.9%	3325
Non-intersection	284	7.9%	682	18.9%	2650	73.3%	3616
*Primary contributing factor*							
Fatigue	29	19.9%	41	28.1%	76	52.1%	146
Speeding	39	12.4%	99	31.5%	176	56.1%	314
Followed too close	21	4.1%	126	24.8%	362	71.1%	509
Improper turn	4	1.1%	32	9.1%	315	89.7%	351
Overcorrection	11	10.4%	29	27.4%	66	62.3%	106
Ran-off-road	37	14.6%	69	27.2%	148	58.3%	254
Ran traffic signal	7	6.9%	40	39.6%	54	53.5%	101
Equipment defect	24	4.8%	54	10.9%	417	84.2%	495
Improper load/size	0	0.0%	3	7.3%	38	1.4%	41
Others	249	5.4%	753	16.3%	3624	78.3%	4626
*First harmful event*							
Collision with a ditch	36	17.7%	57	28.1%	110	54.2%	203
Collision with a guardrail	13	11.8%	23	20.9%	74	67.3%	110
Collision with a tree	23	18.4%	27	21.6%	75	60.0%	125
Others	349	5.4%	1139	17.5%	5017	77.1%	6505
*Most harmful event*							
Rollover	153	15.9%	337	35.0%	473	49.1%	963
Collision with vehicle in traffic	159	4.3%	662	18.0%	2860	77.7%	3681
Others	109	4.7%	247	10.7%	1943	84.5%	2299
*Manner of crash*							
Single vehicle	204	9.4%	453	20.9%	1506	69.6%	2163
Rear-end	81	6.0%	310	22.8%	967	71.2%	1358
Head-on	14	38.9%	6	16.7%	16	44.4%	36
Others	122	3.6%	477	14.1%	2787	82.3%	3386
*Roadway condition*							
Dry	365	6.4%	1054	18.4%	4320	75.3%	5739
Wet	52	5.4%	175	18.2%	737	76.5%	964
Others	4	1.7%	17	7.1%	219	91.3%	240
*Roadway lighting*							
Daylight	306	5.6%	948	17.5%	4163	76.9%	5417
Dark without streetlights	82	9.0%	172	18.9%	654	72.0%	908
Others	33	5.3%	126	20.4%	459	74.3%	618
*Opposing lane separation*							
No separation	26	4.4%	85	14.5%	477	81.1%	588
Concrete barrier	19	2.9%	129	19.6%	510	77.5%	658
Metal/cable barrier	19	4.4%	63	14.5%	352	81.1%	434
Others	357	6.8%	969	18.4%	3937	74.8%	5263
*Development*							
Rural	313	10.1%	610	19.6%	2185	70.3%	3108
Urban	108	2.8%	636	16.6%	3091	80.6%	3835
*Locale*							
Open Country	326	8.9%	736	20.0%	2612	71.1%	3674
Residential	29	4.2%	102	14.8%	556	80.9%	687
Shopping or Business	51	2.4%	335	15.5%	1779	82.2%	2165
Others	15	3.6%	73	17.5%	329	78.9%	417
*Driver residence distance*							
Less than 25 miles	10	4.1%	33	14.2%	202	80.5%	245
More than 25 miles	295	4.4%	969	14.4%	5475	81.2%	6739
*Highway classification*							
Federal	90	7.3%	248	20.1%	894	72.6%	1232
Interstate	70	4.5%	276	17.8%	1205	77.7%	1551
State	146	8.7%	331	19.7%	1204	71.6%	1681
County	85	9.7%	187	21.4%	600	68.8%	872
Others	30	1.9%	204	12.7%	1373	85.4%	1607
*Functional class*							
Interstate	69	4.4%	282	18.1%	1205	77.4%	1556
Minor arterial	98	7.8%	230	18.3%	926	73.8%	1254
Major collector	69	8.7%	171	21.5%	554	69.8%	794
Local	30	4.5%	88	13.2%	551	82.4%	669
Others	155	5.8%	475	17.8%	2040	76.4%	2670
*Time of day*							
Dawn (1 am–6 am)	159	5.7%	486	17.3%	2160	77.1%	2803
Morning (7 am–12 pm)	59	8.5%	151	21.8%	483	69.7%	693
Afternoon (1 pm–6 pm)	46	6.3%	125	17.1%	562	76.7%	733
Night (7 pm–12 am)	157	5.8%	485	17.9%	2071	76.3%	2713

**Table 2:** Descriptive statistics of selected out-of-state large truck crash variables.

Variable	Severe Injury		Minor Injury		No Injury		Total
	Count	Percent	Count	Percent	Count	Percent	Count
*Driver age*							
Less than 25 years old	14	5.1%	38	13.8%	223	81.1%	275
25–40 years old	92	4.3%	304	14.2%	1748	81.5%	2144
41–65 years old	179	4.3%	596	14.2%	3422	81.5%	4197
Above 65 years old	27	6.3%	78	18.2%	323	75.5%	428
*Driver race*							
Caucasian	155	4.4%	511	14.6%	2831	81.0%	3497
Black	110	4.3%	370	14.5%	2075	81.2%	2555
Others	47	4.1%	145	12.6%	962	83.4%	1154
*Crash location*							
Intersection	122	3.6%	485	14.2%	2820	82.3%	3427
Non-intersection	190	5.0%	541	14.3%	3047	80.7%	3778
*Primary contributing factor*							
Fatigue	24	22.9%	25	23.8%	56	53.3%	105
Speeding	24	10.3%	55	23.6%	154	66.1%	233
Followed too close	15	3.1%	106	22.1%	359	74.8%	480
Improper turn	2	1.1%	10	5.7%	162	93.1%	174
Overcorrection	1	2.3%	8	18.6%	34	79.1%	43
Ran-off-road	14	7.9%	28	15.7%	136	76.4%	178
Ran traffic signal	17	14.2%	45	37.5%	58	48.3%	120
Equipment defect	11	2.2%	36	7.1%	461	90.7%	508
Improper load/size	0	0.0%	2	10.0%	18	90.0%	20
Others	190	3.8%	646	12.9%	4166	83.3%	5002
*First harmful event*							
Collision with a ditch	10	12.5%	13	16.3%	57	71.3%	80
Collision with guard rail	10	11.1%	7	7.8%	73	81.1%	90
Others	277	4.2%	934	14.0%	5440	81.8%	6651
*Most harmful event*							
Rollover	43	15.9%	82	30.3%	146	53.9%	271
Collision with vehicle in traffic	143	3.8%	621	16.6%	2970	79.5%	3734
Others	112	3.9%	258	9.0%	2488	87.1%	2858
*Manner of crash*							
Single vehicle	115	6.0%	223	11.6%	1591	82.5%	1929
Rear-end	72	5.6%	269	21.0%	938	73.3%	1279
Others	111	3.0%	469	12.8%	3075	84.1%	3655
*Roadway condition*							
Dry	258	4.6%	813	14.4%	4585	81.1%	5656
Wet	50	4.3%	194	16.7%	919	79.0%	1163
Others	4	1.0%	19	4.9%	364	94.1%	387
*Roadway Lighting*							
Daylight	191	3.9%	703	14.2%	4048	81.9%	4942
Dark without streetlights	80	6.2%	185	14.3%	1031	79.6%	1296
Others	41	4.2%	138	14.3%	789	81.5%	968
*Opposing lane separation*							
No separation	14	2.8%	54	10.7%	439	86.6%	507
Concrete barrier	30	2.6%	177	15.4%	940	82.0%	1147
Metal or cable barrier	26	3.4%	125	16.3%	616	80.3%	767
Others	242	5.1%	670	14.0%	3873	80.9%	4785
*Development*							
Rural	202	6.3%	460	14.3%	2549	79.4%	3211
Urban	96	2.6%	501	13.7%	3054	83.6%	3651
*Locale*							
Open Country	245	6.1%	600	14.8%	3199	79.1%	4044
Residential	10	2.2%	45	10.0%	396	87.8%	451
Shopping or Business	46	2.1%	310	13.9%	1880	84.1%	2236
Others	11	2.3%	71	14.9%	393	82.7%	475
*Driver residence distance*							
Less than 25 miles	14	5.2%	38	14.2%	215	80.5%	267
More than 25 miles	283	4.4%	910	14.1%	5267	81.5%	6460
*Highway classification*							
Federal	69	6.7%	186	18.2%	768	75.1%	1023
Interstate	139	4.6%	439	14.6%	2427	80.8%	3005
State	66	6.4%	164	15.9%	803	77.7%	1033
County	12	3.5%	39	11.4%	292	85.1%	343
Others	12	0.8%	133	9.1%	1314	90.1%	1459
Functional class							
Interstate	146	4.6%	473	14.9%	2552	80.5%	3171
Minor arterial	42	5.7%	98	13.3%	595	81.0%	735
Major collector	14	3.2%	51	11.8%	368	85.0%	433
Local	6	1.4%	31	7.2%	395	91.4%	432
Others	104	4.3%	373	15.3%	1958	80.4%	2435
*Time of day*							
Dawn (1 am–6 am)	92	3.5%	357	13.7%	2158	82.8%	2607
Morning (7 am–12 pm)	70	7.9%	135	15.2%	684	76.9%	889
Afternoon (1 pm–6 pm)	89	4.1%	305	14.2%	1757	81.7%	2151
Night (7 pm–12 am)	48	3.9%	164	13.5%	1004	82.6%	1216

## 3 Methodology

Over the years, highway agencies and vehicle manufacturers have focused on reducing traffic injury severities resulting from motor vehicle crashes. While visible progress has been made in this regard, there still exists the need for additional insights through empirical assessment of the impacts of complex interactions of vehicle, roadway, environment, and human attributes on crash-injury severity outcomes (Savolainen et al., 2011). Traffic researchers have adopted several methodological approaches to uncover the association between crash-injury severity levels and observed and unobserved contributing factors to facilitate the development of effective countermeasures. The recent methodological advances have created robust techniques that accommodate and account for the randomity and unobserved heterogeneity across crash observations. In most cases, the researcher's choice of methodological approach is contingent on the nature of the dependent variable and associated data limitations (Savolainen et al., 2011). Typically, the dependent variable in crash injury severity analysis can be modeled as a binary response outcome (e.g., minor injury or major injury) or multiple response outcomes (e.g., severe injury, minor injury, or no injury). Researchers have also treated response variables with multiple outcomes as either ordinal (ordered) or categorical (unordered) in nature. The application of these various methodological approaches has provided useful insights, but the inherent characteristics of the crash data often result in unaccounted methodological limitations (Savolainen et al., 2011).

Methodological techniques that account for heterogeneity in crash observations are popular within the traffic safety research community. Such approaches include random parameter ordered probability models (Azimi et al., 2020), random parameter multinomial logit models (Ahmadi et al., 2020; Cheng et al., 2019), random parameter models with heterogeneity in means and variances (Behnood and Mannering, 2017; Damsere-Derry et al., 2021), latent class model (Shaheed and Gkritza, 2014), and latent class logit and mixed logit models (Behnood and Mannering, 2016).

Contextually, Guo et al. (2019) used full Bayesian random parameters multivariate Tobit model to examine the correlation and heterogeneity in crash rates by collision types. They found that accounting for heterogeneity improved the model fit significantly in comparison with other Tobit model variants. Random parameter bivariate probit model has been used to examine the impact of driver fatigue, gender and distracted driving on aggressive driving behavior (Fountas et al., 2019). More recent studies have adopted random parameters logit model with heterogeneity in means and variances for traffic crash analysis (Damsere-Derry et al., 2021; Liu et al., 2021; Waseem et al., 2019). Wassem et al. (2019), used random parameters logit model with heterogeneity in means and variances to identify the significant contributing factors to motorcyclists-injury severities in the Pakistian city of Rawalpindi. Damsere-Derry et al. (2021) adopted the same approach to model injury severity of intercity bus crashes in Ghana. In their study Behnood and Mannering (2019) explored the effects of time variation and temporal instability on injury severity in large-truck crashes.

For this study, two separate random parameter multinomial logit models were developed for crashes involving in-state and out-of-state large truck drivers in Alabama while allowing for heterogeneity in the random parameters means and variances. The multinomial logit model is a discrete outcome model that extends the binary logit model to include more than two response outcomes without explicitly considering the existence of potential ordering in the response outcomes. Hence, it is used to model each injury severity class as a categorical response variable with its unique utility function. Marginal effects or pseudo-elasticities are then used to understand the marginal effect of an explanatory variable in one utility function on others. The extended consideration of heterogeneity in means and variances of the random parameters allows for a more generalized approach that captures unobserved heterogeneity across the crash observations (Mannering et al., 2016). For analysis, the response outcomes or dependent variables are usually the various crash or injury severity levels in the data or those modified for the study. Most crash records have five categories of injury severity (i.e., fatal, incapacitating injury, evident injury, minor injury, and no injury) in the data. However, for research purposes, the injury severity levels are often reclassified into three categories, namely, major or severe injury, minor injury, and no injury (Uddin et al., 2020).

While various models can exhibit inherent limitations, the random parameters multinomial logit models with heterogeneity in means and variances approach has produced more statistically superior outcomes in recent crash or injury severity studies. The term crash severity represents the injury level of the most severely injured crash victim. For a crash involving large trucks, the most severely injured victim could be the truck driver or an occupant, another vehicle occupant, or a vulnerable road user. To start with, we define a crash severity function as shown in Eq. (1).

(1) $$S_{kn}=\beta_{k}X_{kn}+\varepsilon_{kn}$$

where $S_{kn}$ is the crash severity function that determines the probability of large truck crash severity category k in crash n, $X_{kn}$ is a vector of explanatory variables that influence the likelihood of large truck crash severity level k in crash n, $\beta_{k}$ is a vector of estimable parameters for crash severity k, and $\varepsilon_{kn}$ is the distributed error term (Washington et al., 2020). If $\varepsilon_{kn}$ is assumed to follow an independent and identically distributed extreme value Type I distribution (McFadden, 1981), and parameter variations are allowed across observations by introducing a mixing distribution. According to McFadden and Train (2000), the resulting mixed logit model is expressed as shown in Eq. (2).

(2) $$P_{n}\left(k\right)=\int \frac{\mathrm{EXP}\left(\beta_{k}X_{kn}\right)}{\sum \mathrm{EXP}\left(\beta_{k}X_{kn}\right)}f\left(\beta \varphi \right)d\beta$$

where f(βφ) is the density function of β with φ representing a vector of parameters of the density function (mean and variance), $P_{n}\;\left(k\right)$ is the probability of crash severity category k in crash n conditioned on f(βφ) and all other terms as previously defined. β can now account for observation-specific variations in the effect of X on crash severity probabilities, with f(βφ) used to determine β. Mixed-logit probabilities are then a weighted average for the different values of β across observations where some elements of the vector β can be fixed or allowed to vary across observations. The parameters that vary across observations are known as random parameters.

The random parameters (mixed) logit models can identify variables that exhibit randomness but cannot account for the factors that explain the randomness in the random variables. We addressed this limitation by adopting random parameters (mixed) logit modeling approach with heterogeneity in means and variances. The technique enables the identification of other variables (within the modeling dataset or from another set of observations) that could further explain the random parameters and their association with the response variables in the presence of other explanatory variables. Therefore, the random parameter (mixed) logit model with heterogeneity in means and variances model can account for unobserved heterogeneity in the dataset than the conventional random parameter models.

The heterogeneity in means and variances of random parameters are modeled according to Eq. (3) by allowing $\beta_{k}$ to vary across crashes (Islam and Mannering, 2020; Waseem et al., 2019).

(3) $$\beta_{k}=\beta +\delta_{k}z_{k}+\sigma_{k}\;\mathrm{EXP}\left(\omega_{k}w_{k}\right)v_{k}$$

where β is the mean parameter estimate across all crashes, $z_{k}$ is a vector of attributes that captures the heterogeneity in the mean, $\delta_{k}$ is the corresponding vector estimable parameters, $w_{k}$ is the vector of attributes that captures heterogeneity in the standard deviation $\sigma_{k}$ with corresponding parameter vector $\omega_{k}$ and a disturbance term $v_{k}$. The vectors $z_{k}$ and $w_{k}$ may contain crash factors or other potential sources of heterogeneity that might be unavailable in the crash database. Model parameters were estimated using simulated maximum likelihood with 1000 Halton draws, which are sufficient for accurate estimation based on previous studies (Anastasopoulos and Mannering, 2009; Bhat, 2003; Halton, 1960; Savolainen, 2016). Additionally, the marginal effects were computed to examine the impact of explanatory variables on the reclassified crash severity outcome probabilities (Washington et al., 2020). By coding all the explanatory variables as indicator variables, the marginal effects are calculated using Eq. (4).

(4) $${ME}_{X_{ijk}}^{P_{ij}}=P_{ij}\left(X_{ijk}=1\right)-P_{ij}\left(X_{ijk}=0\right)$$

The marginal effect of the $k^{th}$ indicator variable, $X_{ijk}$ is the probability difference when $X_{ijk}$ changes from 0 to 1 while other variables are constant. Marginal effects for variables with random parameters across all observations are calculated using only the estimated mean coefficients. Each marginal parameter effect is computed as the average of the marginal effects of all crash observations.

## 4 Model input variables

The outcome of a crash is influenced by several factors usually referred to as the crash contributing factors. For the modeling, the explanatory variables are selected based on the knowledge of significant variables from previous studies on large truck crashes (e.g., Alrejjal et al., 2021; Azimi et al., 2019, 2020; Uddin et al., 2020; Li et al., 2020). The variables cut across driver characteristics, vehicle attributes, and roadway and environmental features. Attention was given to selecting variables that are significant and also improve the model fit. The variable categories included in the final models are briefly described below.

*Primary contributing factor:* This parameter describes the main factor that contributed to the occurrence of the crash. Some common primary contributing factors include speeding, fatigue, overcorrection, and driving under the influence of alcohol or drugs.

*Highway classification:* It describes the type of highway where a crash occurred. The common highway classes are federal, interstate, state, municipal, and county highways. The data shows that most crashes involving in-state and out-of-state drivers were recorded on state and interstate highways, respectively.

*Rural or urban:* This parameter describes the development of the environment where the crash occurred, either as rural or urban.

*First harmful event:* Most crashes are associated with multiple events before the vehicle eventually halts. This parameter describes the preceding event in the crash. In some instances, the first harmful event might not be the most harmful in a crash. Examples of first harmful events include collision with a ditch, collision with a guardrail, and ran-off-road.

*Most harmful event*: This parameter describes the event that seems to contribute mostly to the severity outcome of a crash. It can be the only event in a crash or among the number of events leading to the final crash outcome. Examples include rollover or collision with a vehicle in traffic.

*Opposing lane separation:* This data element describes the separation between the opposing traffic. Examples are concrete barriers (median) and metal/cable barriers.

*Locale:* This variable describes the vicinity of the crash and the common activities in the area. A locale can be a shopping or business area or an open country.

*Driver residence distance:* This variable describes the distance of the crash location from the truck driver's residence. In the data, the distance could either be less than 25 miles or more than 25 miles.

*Road condition:* This parameter describes the condition of the road during the crash. The common road conditions are dry or wet.

*Lighting condition:* This variable describes the lightning state of the road at the time of the crash. Aside from daylight conditions, other lightning conditions include dark with streetlights and dark without streetlights.

*Manner of crash:* This data element describes the form of the crash. Aside from single vehicle large truck crashes, other common “manner of crash” are head-on and rear-end collisions. Head-on or rear-end collisions involves two or more vehicles.

*Road curvature:* This data element describes if a curve on the road contributed to the crash outcome. The road curvatures included in the model are curve left and level and curve right and level.

*Airbag status:* This parameter explains if the vehicle's airbag was deployed or not during the crash. This is a safety related variable and usually have a significant influence on crash outcomes.

*Time of day (TOD):* This variable describes the period when the crash occurred. As shown in the descriptive statistics, TOD was divided into dawn, morning, afternoon, and night.

## 5 Results

Separate random parameter multinomial logit models with heterogeneity in means and variances were estimated for analyzing crashes involving in-state and out-of-state large truck drivers in Alabama. As explained under the data description, three crash severity categories were considered in the models: SI (fatal and incapacitating injury), MI (evident and possible injury), and NI (no injury). The t-statistics of all variables included in the model estimation are statistically significant at a 90% confidence interval or above on a two-tailed t-test. The random parameters were included in the model specification if their standard deviation t-statistics corresponds to a 90% confidence interval or more. Tables 3 and 4 present the detailed model results with their computed marginal effects. The marginal effects indicate the impact of the explanatory variables on the different crash severity categories. From Tables 3 and 4, the McFadden pseudo-$\rho^{2}$ values for the in-state and out-of-state random parameter (mixed) logit models with heterogeneity in means and variances are 0.413 and 0.503, respectively. The McFadden pseudo-$\rho^{2}$ value represents the ratio of the model log-likelihood at constant (model with zero variables except for constants) to the log-likelihood at convergence of the measure (model with all the significant variables). According to Ulfarsson et al. (2010), a $\rho^{2}$ value more than 0.1 indicates meaningful model improvement. Hence, the obtained $\rho^{2}$ values from the two models, as shown in Tables 3 and 4, reflect significant improvement in their goodness-of-fit.

**Table 3:** Model estimation and marginal effects for crashes involving in-state large truck drivers.

Category	Variables	Parameter estimate	t-score	Marginal effects		
				Severe Injury	Minor Injury	No Injury
				[SI]	[MI]	[NI]
*Random parameter*						
Rural or Urban	Rural [SI]	-1.010	-1.45	0.0354	-0.0083	-0.0271
Standard deviation of “Rural” (normally distributed)		2.210	3.58			
*Heterogeneity in means of random parameter*						
Highway Classification	Interstate	-0.570	-1.8			
Driver race	Caucasian	0.492	2.27			
*Heterogeneity in variance of random parameter*						
First harmful event	Collision with a tree	0.631	3.02			
*Defined for severe injury*						
Primary contributing factor	Ran off-road	0.458	1.72	0.0014	-0.0003	-0.0011
First harmful event	Collision with a ditch	0.876	2.68	0.0023	-0.0006	-0.0017
Most harmful event	Rollover	1.332	6.59	0.0163	-0.0041	-0.0122
Intersection	Yes	-0.407	-2.74	-0.006	0.0012	0.0048
Opposing Lane Separation	Metal/cable barrier	-0.725	-2.64	-0.003	0.0006	0.0024
Locale	Shopping or business area	-0.641	-3.44	-0.0042	0.0007	0.0035
Driver residence distance	More than 25 miles	-0.279	-2.08	-0.0062	0.0014	0.0048
Road Condition	Wet	-0.357	-1.76	-0.0018	0.0004	0.0014
Lighting condition	Daylight	-0.858	-5.5	-0.0255	0.0055	0.0201
	Dark without streetlights	-0.660	-2.95	-0.0046	0.0011	0.0035
*Defined for minor severity*						
Primary contributing factor	Fatigue	0.516	2.63	-0.0002	0.0022	-0.002
	Ran traffic signal	1.484	7.06	-0.0001	0.0051	-0.0049
	Overcorrection	0.578	2.4	-0.0002	0.0016	-0.0015
	Swerved to avoid animal	0.626	2.02	-0.0001	0.0009	-0.0008
	Defective equipment	-0.611	-3.99	0.0003	-0.0041	0.0039
Highway Classification	Federal	0.330	3.67	-0.0006	0.009	-0.0085
	County	0.307	2.73	-0.0006	0.0062	-0.0056
Functional class	Major collector	0.196	1.82	-0.0003	0.0036	-0.0033
Locale	Open Country	0.254	3.58	-0.0017	0.0206	-0.0189
Road curvature	Curve left and level	0.347	2.05	-0.0002	0.0019	-0.0017
Manner of crash	Single vehicle	0.447	5.51	-0.0021	0.0222	-0.0201
*Defined for no injury*						
Constant		2.024	24.82			
Primary contributing factor	Improper load/size	1.217	1.95	-0.0001	-0.0004	0.0005
	Ran stop sign	-1.591	-3.46	0.0002	0.0009	-0.0011
Manner of crash	Rear-end	-0.709	-8.97	0.0044	0.0227	-0.027
Highway Classification	State	-0.241	-3.1	0.0024	0.0081	-0.0105
Opposing Lane Separation	Concrete barrier	-0.294	-2.69	0.0006	0.0041	-0.0046
Airbag status	Deployed	0.258	3.94	-0.0032	-0.0146	0.0178
number of observations		6943				
Log-likelihood at convergence		-4480.001				
Log-likelihood at zero		-7627.665				
McFadden Pseudo R-sq		0.413

**Table 4:** Model estimation and marginal effects for crashes involving out-of-state large truck drivers.

Category	Variables	Parameter estimate	t-score	Marginal effects		
				Severe Injury [SI]	Minor Injury [MI]	No Injury [NI]
*Random parameter*						
Lighting condition	Daylight [SI]	-1.953	-2.18	0.0091	-0.0013	-0.0078
*Standard deviation of “Daylight” (normally distributed)*			1.868	2.60		
*Heterogeneity in means of random parameter*						
Primary contributing factor	Improper turning	-1.737	-2.11			
*Heterogeneity in variance of random parameter*						
Most harmful event	Rollover	0.571	3.36			
*Defined for severe injury*						
Driver age	Above 60 years old	-0.409	-1.72	-0.0137	0.0022	0.0116
Primary contributing factor	Speeding	0.899	2.72	0.0018	-0.0003	-0.0015
	Run off-road	0.579	1.7	0.0009	-0.0001	-0.0008
First harmful event	Collision with a ditch	0.763	2.41	0.0014	-0.0002	-0.0012
	Collision with guardrail	0.985	3.38	0.0020	-0.0003	-0.0017
Functional class	Major corridor	-0.787	-2.14	-0.0011	0.0001	0.0010
Opposing Lane Separation	Metal/cable barrier	-0.888	-4.59	-0.0057	0.0009	0.0048
Locale	Shopping or business area	-0.782	-3.78	-0.0043	0.0007	0.0036
Road Condition	Dry	0.400	2.04	0.0111	-0.0018	-0.0093
Airbag status	Deployed	-0.854	-5.1	-0.0093	0.0014	0.0079
Time of day	Night (7 pm–12 am)	-0.429	-2.27	-0.0027	0.0004	0.0023
Season of year	Summer	0.403	2.61	0.0040	-0.0006	-0.0034
*Defined for minor severity*						
Primary contributing factor	Fatigue	1.108	6.58	-0.0005	0.0062	-0.0057
	Ran traffic signal	1.409	7.45	-0.0002	0.0058	-0.0056
	Overcorrection	0.853	3.58	-0.0001	0.0022	-0.0021
	Defective equipment	-0.594	-3.34	0.0001	-0.0029	0.0028
Highway Classification	Federal	0.509	4.55	-0.0005	0.0109	-0.0104
	Interstate	0.417	4.36	-0.0011	0.0226	-0.0215
Road curvature	Curve Left and Level	0.516	2.57	-0.0001	0.0019	-0.0018
Vehicle age	New truck (2010–2020)	-0.272	-3.69	0.0009	-0.0212	0.0202
*Defined for no injury*						
Constant		2.273	20.84			
Manner of crash	Rear-end	-0.566	-6.89	0.0032	0.0165	-0.0197
Most Harmful event	Collision with vehicle in traffic	-0.307	-3.95	0.0041	0.0218	-0.0259
Road curvature	Curve right and level	-0.673	-3.37	0.0006	0.0021	-0.0027
Highway Classification	State	-0.447	-4.23	0.0025	0.0082	-0.0107
	Local	0.633	3.33	-0.0007	-0.0021	0.0028
Number of observations		7206				
Log-likelihood at convergence		-3935.43				
Log-likelihood at zero		-7916.60				
McFadden Pseudo R-sq		0.503				

Two variables were obtained as random parameters for both the in-state and out-of-state drivers’ models. The normal distribution provided the most appropriate fit for the random parameters among the various tested statistical distributions (like normal, lognormal, Weibull, etc.).

For the in-state model, the rural variable (defined for severe injury) was estimated as a random parameter with a mean of −1.010 and a standard deviation of 2.210. These values indicate that with a normal distribution, the variable (rural) is positive for 32.4% of the large truck crash observations (increasing the likelihood of severe injury) and negative for the remaining 67.6% of the observations (decreasing the probability of severe injury). The marginal effect also shows that the rural indicator variable increased the probability of severe injury by 0.0354. Only the interstate and white driver indicators produced significant heterogeneity in means among the explanatory variables. The explanatory variable collision with a tree (as a first harmful event) produced heterogeneity in the variance of the random parameter. The mean of the rural random parameter decreased if the highway classification was interstate, suggesting a decrease in the likelihood of severe injury and an increase in the likelihood of minor and no injury. Ran-off-road, rollover, collision with a ditch, rear-end collision, and collision with vehicle in traffic increase the likelihood of severe injury outcomes. Rollover indicator variable increased the probability of severe injury by 0.0163 marginal points. In daylight condition and at an intersection, the probability of severe injury is significantly decreased by 0.0255 and 0.006 marginal points, respectively. Fatigue, overcorrection, ran traffic light, and single vehicle increase the probability of minor injury. Single vehicle crashes increased the possibility of minor injury by 0.0222 marginal points. For crashes that occurred in an open country, the likelihood of minor injury increases by 0.0206 marginal points.

In the out-of-state model, the daylight variable was estimated as a random parameter (Table 4), having a mean of – 1.953 and a standard deviation of 1.868. These values indicate that the daylight variable decreases the likelihood of severe injury for 85.2% of the crash observations and increases the likelihood of severe injury for the remaining crash observations. Among the explanatory variables, the improper turning (as a primary contributing factor) indicator produced significant heterogeneity in means of the random parameter. The rollover (as a most harmful event) indicator produced significant heterogeneity in the variance of the random parameter. The improper turning indicator variable decreased the mean of the daylight parameter, implying a decrease in the probability of a severe injury and an increase in the probability of minor or no injury. In other words, crashes that involve improper turning in daylight are less likely to result in severe crashes but more likely to result in minor or no injury. Also, in the out-of-state model, speeding, collision with guardrail, dry road condition, and summer increased the probability of severe injury. Older drivers (above 65 years) variable decreased the probability of severe injury but increased the likelihood of minor and no injury.

The explanatory variables, ran-off-road, and collision with a ditch increased the likelihood of severe injury in both models. On the other hand, indicator variables for fatigue, ran traffic signal, overcorrection, federal highway, and road with left curvature and level grade increased the likelihood of minor injury in both models. Table 5 presents a comparison of both model estimates.

**Table 5:** Model results comparison for in-state and out-of-state large truck crashes.

Category	Variable	In-state	Out-of-state
Driver age	>65 years old		↓ SI
Vehicle age	New truck (2010–2020)		↓ MI
Primary contributing factor	Ran off-road	↑ SI	↑ SI
	Fatigue	↑ MI	↑ MI
	Ran traffic signal	↑ MI	↑ MI
	Overcorrection	↑ MI	↑ MI
	Swerved to avoid animal	↑ MI	
	Defective equipment	↓ MI	↓ MI
	Overload	↑ NI	
	Ran stop sign	↓ NI	
	Speeding		↑ SI
First harmful event	Collision with a ditch	↑ SI	↑ SI
	Collision with guardrail		↑ SI
Most harmful event	Rollover	↑ SI	
	Collision with vehicle in traffic		↓ NI
Manner of crash	Single vehicle	↑ MI	
	Rear-end	↓ NI	↓ NI
Intersection	Yes	↓ SI	
Locale	Shopping or business area	↓ SI	↓ SI
	Open Country	↑ MI	
Driver residence distance	More than 25 miles	↓ SI	
Opposing lane separation	Physical separation	↓ SI	↓ SI
	Concrete	↓ NI	
Highway classification	Federal	↑ MI	↑ MI
	Interstate		↑ MI
	County	↑ MI	
	State	↓ NI	↓ NI
	Local		↑ NI
Rural or Urban	Rural	↓ SI	
Road condition	Wet	↓ SI	
	Dry		↑ SI
Lightning condition	Daylight	↓ SI	↓ SI
	Dark without streetlights	↓ SI	
Functional class	Major corridor	↑ MI	↓ SI
Road curvature	Curve Left and Level	↑ MI	↑ MI
	Curve right and level		↓ NI
Airbag status	Deployed	↑ NI	↓ SI
Time of day	Night (7 pm–12 am)		↓ SI
Season of year	Summer		↑ SI

↑ indicates an increase in a severity category, ↓ indicates a decrease in a severity category, SI = severe injury, MI = minor injury, and NI = No injury.

## 6 Model specification test

Researchers have used the Likelihood Ratio (LR) test to determine the suitability of separate models for traffic crash studies (Islam et al., 2014). In this study, an LR test was conducted to justify the estimation of different models for crashes involving in-state and out-of-state large truck drivers using Eq. (5).

(5) $$LR=-2\left[LL\left(\beta_{all}\right)-LL\left(\beta_{in}\right)-LL\left(\beta_{out}\right)\right]$$

where $LL\left(\beta_{all}\right)$ is the log-likelihood at convergence estimated for all the crashes, $LL\left(\beta_{in}\right)$ is the log-likelihood at convergence estimated for crashes involving in-state large truck drivers, and $LL\left(\beta_{out}\right)$ is the log-likelihood at convergence estimated for crashes involving out-of-state large truck drivers. The test statistic is the $\chi^{2}$ distribution having degrees of freedom equal to the sum of the estimated parameters in all the separate models minus the number of estimated parameters in the corresponding model estimated for all crashes. The result showed that the test statistic (LR=116) is more than the corresponding $\chi^{2}$ value of ($\chi^{2}=44.26$) with n degree of freedom (n=15) at a 99.99% confidence interval. We can then reject the null hypothesis that the combined model log-likelihood is not significantly different from the corresponding separate models and conclude that a separate model specification is suitable.

## 7 Discussion

Freight transport contributes significantly to the overall economic development of a state (Wang et al., 2021). A prerequisite for this, however, is the timely arrival of goods at their destinations. Truck-involved crashes are one of the leading causes of disruption to the timely delivery of goods. As such, much effort has been devoted to understanding the factors that contribute to truck-involved crashes and crash outcomes. Considering that crash incident clearance time is significantly impacted by the crash severity (Ding et al., 2015; Islam et al., 2021; Ji et al., 2014), this study contributes to this effort by exploring differences and similarities in crashes involving in-state and out-of-state truck drivers. It is worth noting that a preliminary analysis of the data used in the study showed similar proportions of crashes involving in-state and out-of-state drivers across the years. This indicates that irrespective of truck drivers’ state of residence, the factors contributing to their crashes are perhaps generally very similar.

However, viewing large truck crashes through the lens of the driver's state of residence is particularly important as familiarity with the roadway and driver fatigue are major contributing factors to crashes. Indeed, Figure 3 revealed a clear difference in the proportion of fatigue-related crashes between in-state and out-of-state drivers. Similarly, it was observed that out-of-state drivers were involved in more crashes related to running traffic signals. These findings point to the fundamental issues of fatigue among long distance truck drivers and their unfamiliarity with the operations of signalized intersections in Alabama. Speeding was found to contribute to more crashes among in-state drivers. In-state drivers might be more familiar with the roadway characteristics than their out-of-state counterparts. However, such familiarity could encourage unsafe actions like speeding, possibly compromising traffic safety. Figure 2 further revealed that the injury outcomes of truck-involved crashes are not significantly different between in-state and out-of-state drivers.

To better understand the crash factors that significantly influence crash outcomes, two separate injury-severity models were estimated. For ease of comparison, Table 5 was developed to present the variables significantly contributing to large truck-involved crash outcomes between in-state and out-of-state drivers. From Table 5, it can be observed that the factors that influence large truck crash outcomes do not significantly differ between in-state and out-of-state drivers. This finding is interesting because, whereas different factors may contribute to the occurrence of crashes, the outcome of the crashes are similar. Perhaps, this may be more related to the vehicle characteristics (e.g., age, size, configuration, etc.) and less to driver demographics. However, it should be noted that even though the crash factors have similar effects on crash outcomes, they do so to varying degrees. For instance, for the collision with a ditch variable, the marginal effects indicate that this variable increases the probability of severe injury by 0.0023 and 0.0014 in the in-state and out-of-state models, respectively. This difference in magnitude shows that truck crashes involving in-state drivers are more likely to result in severe injury compared to out-of-state drivers. There were more speeding related crashes among the in-state large truck drivers, but the contribution of speeding to minor crash severity outcomes was only significant in the out-of-state model. It is quite interesting that speeding was not significant in the in-state model as one would have expected. However, since speeding contribute to significant proportion of the crashes, measures should be taken to adequately enforce speeding regulations.

In this study, it was observed that fatigue and red light running were associated with increased crash severity in both in-state and out-of-state truck large truck crashes. These findings are generally consistent with previous studies (Azimi et al., 2019; Hosseinzadeh et al., 2021). Contrary to Islam and Hernandez (2013), the results of this study revealed that summer season was more likely to be linked with severe injury outcomes for crashes involving out-of-state large truck drivers. FHWA (2020) made an interesting finding that summer months are associated with vehicle overheating and traffic congestion and these usually result in vehicle malfunctioning and increased traffic incidents. Indeed, statistics have also shown that severe road crashes involving large trucks are more likely in summer compared to other seasons (Ahmed et al., 2020).

Despite the seeming similarity in factors that influence crash outcomes, this study provides the basis for truck driver training and awareness creation on differences that may exist in roadway characteristics from state to state. Communication campaigns should be incorporated with training programs as evidence has shown that simultaneously implementing both measures is more effective (Faus et al., 2021). Long-distance truck drivers should be encouraged to use rest areas to reduce the likelihood of getting into fatigue-related crashes (Adanu et al., 2021a, Adanu et al., 2021b; Pulugurtha et al., 2022). Driver-assist technologies also have the potential to complement the driving task for truck drivers, hence minimizing the chances of getting into crashes. Regular sensitization and adequate traffic law enforcement are necessary to ensure road safety compliance among truck drivers. In the long term, this is expected to promote a positive safety culture among truck drivers. Policy formulations and strategies that prioritize truck drivers’ well-being and create better working conditions for them should be explored. Also, exchange driver(s) should be provided for long-distance trips to minimize fatigue driving and keep the main driver engaged.

## 8 Conclusion

This study extends the understanding of truck-involved crashes by examining in-state and out-of-state large truck crashes in Alabama. The analysis was based on 2016–2020 large truck crashes obtained from the Critical Analysis Reporting Environment (CARE) developed by the University of Alabama Center for Advanced Public Safety. Crashes involving in-state and out-of-state drivers were classified based on the driver's state of residence. Crashes involving truck drivers residing in Alabama were classified as in-state and others as out-of-state. Two random parameter logit models with heterogeneity in means and variances were estimated to identify the significant contributing factors to the severity outcomes of crashes involving in-state and out-of-state drivers. An initial descriptive analysis of the data revealed that speeding contributes to more in-state crashes and running traffic light contributes to more out-of-state crashes. Of all the primary contributing factors, fatigue contributed to a significant proportion of the crashes and more for crashes involving out-of-state drivers.

The model results revealed that the crash contributing factors do not differ significantly among in-state and out-of-state drivers. Factors like fatigue, ran off-road, ran traffic signal, overcorrection, and collision with a ditch significantly increased severity outcomes in both models. Speeding was associated with more in-state crashes but was only significant in the out-of-state crash model. Perhaps, familiarity with the roadway among in-state drivers encourage more speeding but is probably less likely to result in severe crash outcomes. Also, overcorrection was predominant among in-state drivers. However, overcorrection increased the probability of minor injury for crashes involving out-of-state crashes by 0.0022 marginal points compared to 0.0016 for in-state drivers. Interstate, dry road condition, and summer season are other factors that significantly increase severe or minor injury outcomes for crashes involving out-of-state drivers.

Regarding heterogeneity, the impact of rural areas and daylight lightning conditions showed significant variations among crash observations involving in-state and out-of-state drivers, respectively. This suggests that truck-involved crashes in rural areas or under daylight conditions are less likely to result in severe injury outcomes. However, the presence of other variables like fatigue, speeding, or running traffic light could increase the injury severity outcomes. Besides the theoretical advantages of this study, the consideration of heterogeneity across the crash observations ensured better model estimations and proposal of efficient countermeasures. Compared to the existing literature on severity analysis of truck-involved crashes, this study identified similar significant contributing factors. Consistent with prior studies, factors such as fatigue, ran off-road, overcorrection, running red light, speeding, collision with vehicle in traffic, dry road condition, curve left and level, and summer season increased the probability of severe injury outcomes. The study also identified that single vehicle and rollover crashes involving in-state large truck drivers in open country are associated with an increased likelihood of severe outcomes. Despite the seeming significant similarity in factors that influence crash outcomes, this study provides the basis for truck driver training and awareness creation on differences that may exist in roadway characteristics from state to state.

In spite of the findings of this study which are interesting and consistent with previous works, there are few limitations that should be noted. The quality of any statistical model depends on the quality of the data used. Underreporting is a common limitation of crash data (Kayani et al., 2014; Watson et al., 2015), especially for minor or no injury crashes. Crash reporting officers usually rely on their discretion, drivers, and eyewitness accounts to determine the contributing factors in a crash. The collected information, based on a standard reporting system format, might not accurately represent all contributing factors. Where the driver refused to disclose some information regarding the crash, the reporting officer would have to rely on their experience to make a decision. These discrepancies can create “bias” in the dataset, usually called unobserved heterogeneity. However, in the absence of other sources of crash information, researchers often rely on police-reported crash records and adopt statistical methods to account for some of the unobserved heterogeneity in the data. In that regard, this study developed a random parameter multinomial logit model with heterogeneity in the means and variances. Traffic volume during the crash was not available as an input variable for this study. As a contributing factor, traffic volume could improve the model and provide additional insights into the difference between crashes involving in-state and out-of-state drivers. The classification of in-state or out-of-state drivers was based on the driver's license state (i.e., the state that issued the driving license) regarded as the state of residence. The assumption was that most drivers reside in the state where their driving license was issued. This approach might not be accurate given that some drivers might obtain their license in one state but reside or work in the state where the crash occurred. This possible interlink within the data could create a distinctive classification challenge. Based on the study outcome and highlighted limitations, the following suggestions are provided for future research direction.

• Future studies should explore better ways to classify crashes involving in-state and out-of-state drivers by interpolating two or more variables. For instance, if the truck driver's origin-destination is known (which was not available for this study), a better classification of in-state and out-of-state crashes could be obtained by interpolating it with the driver state of residence considered as the driver's license state in this study.
• The imbalance in the dataset, as shown in Figure 2, where observations from a particular injury severity class have a significantly lower frequency than the other classes, could present a challenge of inadequate variability in model estimations. Therefore, future works should compare different modeling techniques including machine learning to identify the method (s) with improved model performance.
• The study could benefit from additional information from a survey of truck drivers for countermeasures proposition. Therefore, future studies can incorporate a survey of truck drivers in and outside Alabama to understand the challenges of the drivers that might be critical to traffic safety.

## Declarations

### Author contribution statement

Sunday Okafor: Conceived and designed the experiments; Performed the experiments; Analyzed and interpreted the data; Wrote the paper.

Emmanuel Kofi Adanu: Analyzed and interpreted the data; Contributed reagents, materials, analysis tools or data; Wrote the paper.

Steven Jones: Contributed reagents, materials, analysis tools or data; Wrote the paper.

### Funding statement

This research did not receive any specific grant from funding agencies in the public, commercial, or not-for-profit sectors.

### Data availability statement

Data will be made available on request.

### Declaration of interest's statement

The authors declare no conflict of interest.

### Additional information

No additional information is available for this paper.

## References

[bib1] Adanu E.K., Hu Q., Liu J., Jones S. (2021). Better rested than sorry: data-driven approach to reducing drowsy driving crashes on interstates. J. Transport. Eng., Part A: Systems.

[bib2] Adanu E.K., Lidbe A., Tedla E., Jones S. (2021). Injury-severity analysis of lane change crashes involving commercial motor vehicles on interstate highways. J. Saf. Res..

[bib3] Ahmadi A., Jahangiri A., Berardi V., Machiani S.G. (2020). Crash severity analysis of rear-end crashes in California using statistical and machine learning classification methods. J. Transport. Saf. Secur..

[bib4] Ahmed I.U., Gaweesh S.M., Ahmed M.M. (2020). Exploration of hazardous material truck crashes on Wyoming’s interstate roads using a novel Hamiltonian Monte Carlo Markov chain bayesian inference. Transport. Res. Rec.: J. Transport. Res. Board.

[bib5] Alrejjal A., Farid A., Ksaibati K. (2021). A correlated random parameters approach to investigate large truck rollover crashes on mountainous interstates. Accid. Anal. Prev..

[bib6] Anastasopoulos P.C., Mannering F.L. (2009). A note on modeling vehicle accident frequencies with random-parameters count models. Accid. Anal. Prev..

[bib7] Apostolopoulos Y., Sönmez S., Shattell M.M., Belzer M. (2010). Worksite-induced morbidities among truck drivers in the United States. AAOHN J..

[bib8] Azimi G., Asgari H., Rahimi A., Jin X. (2019). Transportation Research Board 98th Annual Meeting.

[bib9] Azimi G., Rahimi A., Asgari H., Jin X. (2020). Severity analysis for large truck rollover crashes using a random parameter ordered logit model. Accid. Anal. Prev..

[bib10] Behnood A., Mannering F. (2017). The effect of passengers on driver-injury severities in single-vehicle crashes: a random parameters heterogeneity-in-means approach. Anal. Methods Accid. Res..

[bib11] Behnood A., Mannering F. (2019). Time-of-day variations and temporal instability of factors affecting injury severities in large-truck crashes. Anal. Methods Accid. Res..

[bib12] Behnood A., Mannering F.L. (2016). An empirical assessment of the effects of economic recessions on pedestrian-injury crashes using mixed and latent-class models. Anal. Methods Accid. Res..

[bib13] Bhat C.R. (2003). Simulation estimation of mixed discrete choice models using randomized and scrambled Halton sequences. Transp. Res. Part B Methodol..

[bib14] Cheng L., Caset F., de Vos J., Derudder B., Witlox F. (2019). Investigating walking accessibility to recreational amenities for elderly people in Nanjing, China. Transport. Res. Transport Environ..

[bib15] Damsere-Derry J., Adanu E.K., Ojo T.K., Sam E.F. (2021). Injury-severity analysis of intercity bus crashes in Ghana: a random parameters multinomial logit with heterogeneity in means and variances approach. Accid. Anal. Prev..

[bib16] Ding C., Ma X., Wang Y., Wang Y. (2015). Exploring the influential factors in incident clearance time: disentangling causation from self-selection bias. Accid. Anal. Prev..

[bib17] Duffin E. (2022).

[bib18] Faus M., Alonso F., Fernández C., Useche S.A. (2021). Are traffic announcements really effective? A systematic review of evaluations of crash-prevention communication campaigns. Saf. Now..

[bib19] FHWA (2020). https://ops.fhwa.dot.gov/congestion_report/chapter2.htm.

[bib20] Fountas G., Pantangi S.S., Hulme K.F., Anastasopoulos P.C. (2019). The effects of driver fatigue, gender, and distracted driving on perceived and observed aggressive driving behavior: a correlated grouped random parameters bivariate probit approach. Anal. Methods Accid. Res..

[bib21] Garbarino S., Guglielmi O., Sannita W., Magnavita N., Lanteri P. (2018). Sleep and mental health in truck drivers: descriptive review of the current evidence and proposal of strategies for primary prevention. Int. J. Environ. Res. Publ. Health.

[bib22] Guo Y., Li Z., Liu P., Wu Y. (2019). Modeling correlation and heterogeneity in crash rates by collision types using full bayesian random parameters multivariate Tobit model. Accid. Anal. Prev..

[bib23] Halton J.H. (1960). On the efficiency of certain quasi-random sequences of points in evaluating multi-dimensional integrals. Numer. Math..

[bib24] Heaton K. (2005). Truck driver hours of service regulations: the collision of policy and public health. Pol. Polit. Nurs. Pract..

[bib25] Hosseinzadeh A., Moeinaddini A., Ghasemzadeh A. (2021). Investigating factors affecting severity of large truck-involved crashes: comparison of the SVM and random parameter logit model. J. Saf. Res..

[bib26] Islam M., Hernandez S. (2013). Large truck–involved crashes: exploratory injury severity analysis. J. Transport. Eng..

[bib27] Islam M., Mannering F. (2020). A temporal analysis of driver-injury severities in crashes involving aggressive and non-aggressive driving. Anal. Methods Accid. Res..

[bib28] Islam N., Adanu E.K., Hainen A.M., Burdette S., Smith R., Jones S. (2021). A comparative analysis of freeway crash incident clearance time using random parameter and latent class hazard-based duration model. Accid. Anal. Prev..

[bib29] Islam S., Jones S.L., Dye D. (2014). Comprehensive analysis of single- and multi-vehicle large truck at-fault crashes on rural and urban roadways in Alabama. Accid. Anal. Prev..

[bib30] Ji Y., Jiang R., Qu M., Chung E. (2014). Traffic incident clearance time and arrival time prediction based on hazard models. Math. Probl Eng..

[bib31] Kayani A., Fleiter J.J., King M.J. (2014). Underreporting of road crashes in Pakistan and the role of fate. Traffic Inj. Prev..

[bib32] Li J., Liu J., Liu P., Qi Y. (2020). Analysis of factors contributing to the severity of large truck crashes. Entropy.

[bib33] Liu P., Fan W. (David) (2022). Analyzing injury severity of rear-end crashes involving large trucks using a mixed logit model: a case study in North Carolina. J. Transport. Saf. Secur..

[bib34] Liu S., Fan W.D., Li Y. (2021). Injury severity analysis of rollover crashes for passenger cars and light trucks considering temporal stability: a random parameters logit approach with heterogeneity in mean and variance. J. Saf. Res..

[bib35] Mannering F.L., Shankar V., Bhat C.R. (2016). Unobserved heterogeneity and the statistical analysis of highway accident data. Anal. Methods Accid. Res..

[bib36] McFadden D. (1981).

[bib37] McFadden D., Train K. (2000). Mixed MNL models for discrete response. J. Appl. Econom..

[bib38] National Safety Council (2022). https://injuryfacts.nsc.org/motor-vehicle/road-users/large-trucks/.

[bib39] NHTSA (2022). https://www-fars.nhtsa.dot.gov/Vehicles/VehiclesAllVehicles.aspx.

[bib40] NHTSA (2022). https://crashstats.nhtsa.dot.gov/Api/Public/ViewPublication/813266.

[bib41] Pahukula J., Hernandez S., Unnikrishnan A. (2015). A time of day analysis of crashes involving large trucks in urban areas. Accid. Anal. Prev..

[bib42] Placek M. (2022).

[bib43] Pulugurtha S.S., Duvvuri S., Mathew S. (2022).

[bib45] Savolainen P.T. (2016). Examining driver behavior at the onset of yellow in a traffic simulator environment: comparisons between random parameters and latent class logit models. Accid. Anal. Prev..

[bib46] Savolainen P.T., Mannering F.L., Lord D., Quddus M.A. (2011). The statistical analysis of highway crash-injury severities: a review and assessment of methodological alternatives. Accid. Anal. Prev..

[bib47] Shaheed M.S., Gkritza K. (2014). A latent class analysis of single-vehicle motorcycle crash severity outcomes. Analytic Methods in Accident Research.

[bib48] Tahfim S.A.-S., Yan C. (2021). Analysis of severe injuries in crashes involving large trucks using K-prototypes clustering-based GBDT model. Saf. Now..

[bib49] Uddin M., Huynh N. (2020). Injury severity analysis of truck-involved crashes under different weather conditions. Accid. Anal. Prev..

[bib50] Ulfarsson G.F., Kim S., Booth K.M. (2010). Analyzing fault in pedestrian–motor vehicle crashes in North Carolina. Accid. Anal. Prev..

[bib51] Wang H., Han J., Su M., Wan S., Zhang Z. (2021). The relationship between freight transport and economic development: a case study of China. Res. Transport. Econ..

[bib52] Waseem M., Ahmed A., Saeed T.U. (2019). Factors affecting motorcyclists’ injury severities: an empirical assessment using random parameters logit model with heterogeneity in means and variances. Accid. Anal. Prev..

[bib53] Washington S., Karlaftis M., Mannering F., Anastasopoulos P. (2020).

[bib54] Watson A., Watson B., Vallmuur K. (2015). Estimating under-reporting of road crash injuries to police using multiple linked data collections. Accid. Anal. Prev..

